# The Use of a Ward Round Teaching Tool in a Paediatric Oncology Department

**DOI:** 10.1111/jpc.70155

**Published:** 2025-07-17

**Authors:** Trisha A. Soosay Raj, Natacha Omer, Amy Z. Gray

**Affiliations:** ^1^ Department of Oncology Queensland Children's Hospital Brisbane Australia; ^2^ University of Melbourne Melbourne Australia; ^3^ University of Queensland Brisbane Australia; ^4^ Department of Paediatrics Royal Children's Hospital Melbourne Australia

**Keywords:** clinical learning environment, education, framework, learner, Ward rounds

## Abstract

**Aim:**

Despite ward rounds being fundamental to hospital‐based clinical training, the reported educational value is low. Especially in busy environments, missed learning opportunities occur due to implicit learning, time barriers, and lack of ward round structure. The STIC framework (*S*et, *T*arget, *I*nspect, *Close*) provides a learner‐centred, structured approach to ward round teaching, aimed to enhance education within limited timeframes. We aimed to investigate how the introduction of the STIC framework impacts on learner‐centred teaching within a tertiary Paediatric Oncology department.

**Methods:**

A mixed‐methods approach was used to evaluate framework implementation, with two participant groups comprising 16 junior and senior doctors over 3 months. Surveys were used to document junior staff experience on rounds pre‐ and post‐implementation, with focus groups and interviews used for all participants to explore satisfaction and attitudes to the tool.

**Results:**

Learner satisfaction improved across all domains of the framework, specifically opportunities for leading clinical encounters and learning on consultant‐led rounds. Despite consultants reporting lack of uptake, trainees reported improved teaching. Consultant beliefs and enthusiasm had a strong impact on trainee satisfaction. Trainees placed a high value on active participation and autonomy for their learning. Factors distinct to teaching were reported to affect learner satisfaction, such as planning, time management, and departmental culture.

**Conclusions:**

We demonstrate enhanced teaching despite poor perceived uptake, demonstrating the potential of the STIC framework with further implementation. Our study also highlights that in addition to a specific teaching focus, consultant engagement and a safe clinical learning environment are crucial for learning.

**Summary**

- What is already known on this topic
  ○ The educational value of ward rounds is low, despite being an integral component of hospital‐based training for junior doctors.
  ○ Resource limitations and lack of ward round structure lead to missed learning opportunities, disorganisation, inadequate role definition, and reduced trainee satisfaction.
  ○ Learners more commonly have passive roles in the clinical environment despite evidence of the benefits of active participation and learner‐centred teaching.
- What this paper adds
  ○ Consultants have a strong influence; small changes in consultant engagement and effort can significantly improve trainee satisfaction with ward round learning.
  ○ A ward round teaching tool has the potential to improve learner satisfaction with teaching, including enhanced supported independence and feedback provision.
  ○ The clinical learning environment, including departmental culture and psychological safety, is a crucial factor for trainee satisfaction with learning.

## 1 Introduction

Ward rounds are a key clinical component in hospitals and are recognised as entrustable professional activities for trainee doctors [1]. However, their reported educational value is low [2, 3], further impacted in busy departments, with inadequate time reported as the greatest teaching barrier across all levels [3].

Ward‐based teaching is mostly consultant‐led, with passive learner roles [2, 3, 4, 5]. Learning is often reported to be implicit, particularly communication and giving feedback [6]. Missed learning opportunities and the hidden curriculum involve assumed hierarchy, implicit role allocation, and deficient ward round structure [2, 4, 6]. The ineffectiveness of didactic teaching highlights the importance of active learning [7]. Keeping with social constructivism, teaching should prioritise learners, including taking lead roles, with increased satisfaction reported using learner‐identified topics [8].

Despite ward rounds being instrumental to learning, resourcing and educational support is limited for consultants to implement this complex task, with resulting service provision emphasis rather than teaching [1]. Lack of ward round structure correlates with varied learning opportunities, disorganisation, inadequate role clarity and reduced trainee satisfaction [6]. The STIC framework provides a learner‐centred, structured approach to ward round teaching [9]. This framework comprises four components: Set (agenda and roles), Target (teaching), Inspect (reflection and feedback) and Close (closure of clinical and learning) [9]. Despite extensive literature on ward round teaching barriers [2, 3, 4, 5], evidence is limited about potential interventions like ward round tools for teaching [10].

We aimed to investigate how introducing the STIC framework impacts on learner‐centred teaching within a tertiary Paediatric Oncology department.

## 2 Methods

### 2.1 Study Design

This single‐centre mixed‐methods study evaluated the implementation of the STIC tool, with two non‐randomised participant groups comprising junior and senior doctors (consultants) [9]. This study draws from social constructivism, with social interactions and learner roles within teams essential to knowledge building [11]. An equal‐status sequential explanatory design with follow‐up explanation variant was used, collecting quantitative and qualitative data at different timepoints [12]. Implementation comprised videos, leaflets, and email reminders for all participants. Quantitative data collection comprised surveys for junior staff 4 weeks pre‐ and 4 weeks post‐implementation, using 5‐point Likert scale measurements from 1 (most negative) to 5 (most positive). Additional data were collected using focus groups to expand on survey results and allow triangulation of data. Consultants were invited to a focus group at study completion exploring satisfaction and attitudes to the tool.

### 2.2 Setting

This study was conducted in the Oncology department of an Australian tertiary paediatric hospital, encompassing 11 consultants and 9 junior doctors across two medical teams. The junior staff comprise four resident medical officers (post‐graduate year[PGY] 3), three registrars (usually PGY4) and two oncology fellows (PGY5 and above). Daily ward rounds are led by a consultant or fellow. The study was conducted from August to October 2020, scheduled with a resident rotation for continuity.

### 2.3 Recruitment

All departmental medical staff participating in rounds during the study period was eligible. Purposive sampling was used, inviting all medical staff and identifying seconded junior doctors at orientation. Participants received a verbal presentation and written plain language statement. Consent was obtained verbally for surveys and written for focus groups. Junior staff participants could choose to enrol in both surveys and focus groups, or only one component. As the lead researcher had a dual role as a consultant, a co‐researcher supported recruitment to prevent coercion.

### 2.4 Data Collection

Surveys targeted assessment of learner satisfaction with ward rounds across STIC domains. Surveys were anonymous, paper‐based, and brief using 5‐point Likert scales [12]. They comprised six closed‐ended questions and were performed daily due to the variable nature of ward rounds (Figure S1). Post‐STIC surveys included documentation of participants' clinical teams based on interim qualitative results indicating potential differences. Surveys were stored securely.

Focus groups of junior and senior doctors were planned to further evaluate participants' perception of the educational value of ward rounds and attitudes to teaching and learning (Table S1). These were ultimately performed as a combination of focus groups (four) and face‐to‐face interviews (four) to facilitate attendance, lasting 30–60 min, using semi‐structured interview guides and facilitated by the lead researcher. Discussions were recorded via a secure digital voice recorder and subsequently deleted. Transcriptions were performed by the lead researcher, with data de‐identified and stored securely.

### 2.5 Data Analysis

Quantitative data were analysed using descriptive and inferential statistics. The mean Likert Scale ratings and percentage agreeing with each survey statement were calculated. To compare outcomes pre‐ and post‐implementation, independent *t* tests were used to compare means and *χ* ^2^ test of independence to compare percentages responding in each category (using SPSS Version 26.0).

Qualitative data were analysed using inductive content analysis on NVivo (NVivo software, V.12) to identify codes and create themes. This was done iteratively, with themes identified by the primary researcher and verified by a co‐researcher. This data was triangulated with quantitative outcomes to understand impact.

The primary outcome measure was the overall mean junior staff rating of learner‐centred wards round teaching. Secondary outcome measures comprised mean ratings of components of learner‐centred teaching and themes regarding broader effects of ward round teaching on team performance and consultant satisfaction after STIC.

This study was approved by the Children's Health Queensland Hospital and Health Service Human Research Ethics Committee (Reference: LNR/20/QCHQ/61830).

## 3 Results

A total of 16 participants contributed to this study, comprising nine junior doctors and seven consultants. Oncology fellows completed surveys only when attending ward rounds led by a consultant and were interviewed separately as frequent ward round leaders. All staff participated for the entire study period, with all junior doctors participating. Among consultants, one was a researcher, thus not included in data collection, and three were invited but not able to participate in focus groups or interviews, with 64% (7/11) participating.

Pre‐implementation, 87 surveys were collected from junior doctors, and 89 post‐implementation (Table 1).

**TABLE 1:** Demographic data.

Participant hierarchy	No.	Surveys pre‐STIC	Surveys post‐STIC	Focus groups (no. of participants)	Interviews
Junior medical officer	4	87	89	1 (4)	—
Registrar	3			1 (2)	1
Fellow	2			—	2
Consultant	7	—	—	2 (3)	1

*Note*: Demographic data of participants demonstrating hierarchy, numbers of participants, surveys completed pre‐ and post‐implementation of the STIC tool, focus group and interview numbers and participants.

Abbreviation: No., number.

Baseline mean learner satisfaction for overall ward round learning was 3.05 (range 1–5). Feedback provision had the lowest mean score, (2.32 (range 1–5)), followed by setting the agenda (2.65; range 1–5) and closing the round (2.85, range 1–5). The highest mean score (3.25) related to opportunities to lead during ward rounds (range 1–5). One respondent was an outlier, documenting ‘strongly disagreed’ to all questions and noting they were performing tasks for the entire ward round.

**FIGURE 1:**
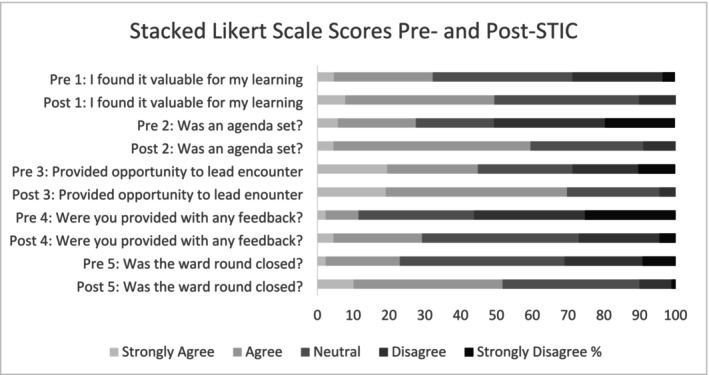
Stacked Likert Scale scores pre‐ and post‐STIC. Stacked Likert Scale scores demonstrating junior doctor perceptions of the educational value and activities of ward rounds, pre‐ and post‐implementation of the STIC tool.

There was an improvement in mean Likert scores across all domains in the post‐implementation period, with a mean absolute effect size of 0.64 (*p* < 0.005) (Table 2 and Figure 1).

**TABLE 2:** Survey results pre‐ and post‐STIC.

Question	Pre‐STIC mean	95% CI	Range (min‐max)	Post‐STIC mean	95% CI	Range (min–max)	Mean diff.	*p*
Overall learning	3.05	2.85–3.24	1–5	3.45	3.27–3.62	1–5	0.40	0.003
Setting of agenda	2.65	2.40–2.90	1–5	3.49	3.32–3.64	2–5	0.84	< 0.001
Opportunities to lead	3.25	2.99–3.52	1–5	3.84	3.68–4.00	2–5	0.59	< 0.001
Provided with feedback	2.32	2.11–2.54	1–5	3.01	2.83–3.21	1–5	0.69	< 0.001
Closing of ward round	2.85	2.65–3.05	1–5	3.51	3.33–3.68	1–5	0.66	< 0.001

*Note*: Mean Likert scores with 95% confidence interval, range and *p* value, demonstrating junior doctor perceptions of the educational value and activities of ward rounds, pre‐ and post‐implementation of the STIC tool.

Abbreviations: 95% CI, 95% confidence interval; max, maximum; mean diff, mean difference; min, minimum.

Ward rounds were more frequently led by consultants or fellows post‐implementation (69% pre‐ and 80% post‐STIC). Despite this increase in senior leadership, participants reported improved opportunities to lead clinical encounters (3.25 pre‐ and 3.84 post‐STIC (*p* < 0.005)). Junior staff reported the highest learning value from self‐led rounds, with mean scores of 3.9 pre‐ and 3.75 post‐STIC. No junior doctor disagreed with the statement ‘I found the ward round valuable to my learning’ for self‐led rounds. For consultant‐led rounds, the proportion of respondents disagreeing to the same statement decreased from 47% pre‐ to 7% post‐STIC (*p* < 0.005).

Across the two clinical teams, overall learner satisfaction was higher post‐STIC in one team, with a mean score of 3.16 versus 3.64 (*p* = 0.017), with comparable satisfaction between teams otherwise.

During focus groups a clear theme emerged of a perceived lack of uptake of the STIC tool among senior medical staff, leading to a discussion regarding general attitudes towards teaching. Although junior staff demonstrated a clearer understanding and enthusiasm for the tool, they similarly focused on broader aspects of ward‐based teaching.

We identified three major themes from the qualitative data that impacted learner satisfaction on ward rounds: Consultant Influence, Supported Independence, and the Clinical Learning Environment (Table 3).

**TABLE 3:** Themes and quotes from focus groups and interviews.

Theme	Subtheme	Illustrative quotes
Consultant influence	Engagement	‘My view is the main purpose of the ward round is to make sure the patients are okay. Then there is also a teaching component to it. But my first priority is to sort of fix the patients’ *Consultant* ‘There wasn't a lot of time for formal teaching, but one would hope that the students were observant. And they should have learned a lot’ *Consultant* ‘think it worked by being stuck in people's head as a reminder to focus more on teaching’ *Trainee* ‘There was an active effort from the consultants and fellows to sort of having teaching points each day. So I found there was some penetrance of the project’ *Trainee*
	Individual and team influence	‘Our experience was very different to you guys. Most of my ward rounds were not really, like focused on teaching, they're very task‐focused’ *Trainee* ‘There were definitely a few weeks when nearly every single day I rounded by myself. Which was good from our point of view of making you a bit more independent. But I think it needed to be a bit more mixed. Because I wasn't getting any teaching’ *Trainee*
Supported independence	Active learning	‘The fellow did quite a good job of teaching you something and then being like, right, now you're doing it with the parents. That was actually a really good learning exercise’ *Trainee* ‘I actually hate being observed doing stuff, but it's what makes me learn the best’ *Trainee*
	Autonomy	‘For me, that's a big one (seeing patients by myself) …they have significant clinical findings and sometimes it might be nice to actually discuss that’ *Trainee* ‘It stops us from being able to transition, and suddenly you're supposed to be able to lead the round, …, how are you actually going to know what to do?’ *Trainee*
Clinical learning environment	Management	‘I genuinely don't think that in the type of week (we have), I mean, it's damage control’ *Consultant* ‘If they round at the end of the day, then you just do a quick round and more for patient safety rather than learning’ *Trainee*
	Culture	‘You get a bit like scared to say anything. And then I just hold back and wait to say to someone else, …, where you wouldn't get shut down for not understanding it’ *Trainee* ‘And even interact with the patients cause I feel sometimes they don't know who you are, because you're standing behind a computer all the time. And then when you do evenings, or you do weekends, they're like “who are you?” And you're like, “oh, actually, I've been seeing you for like three weeks now. Behind the computer, so you don't really see me? I don't really talk.” Yeah, it's really sad’ *Trainee*

*Note*: Themes, subthemes, and quotations from qualitative data on the impact of the STIC framework on teaching and learning on junior and senior medical staff, barriers to ward round teaching and learning, and consultant attitudes toward round teaching.

### 3.1 Consultant Influence

Trainees reported a strong consultant influence on frequency and quality of ward round teaching. Consultants demonstrating active teaching effort and enthusiasm improved learner satisfaction. Individual consultant beliefs impacted teaching, for example the role of a ward round for patient management or teaching. Attitudes to learning methods impacted teaching, with one consultant reporting passive observation as an expected learning method. There were also differing opinions regarding the role of feedback; as correction, positive praise or a teaching tool. Reflecting survey findings, junior staff reported contrasting learning experiences across clinical teams, highlighting an additional consultant influence at team level. In the team with few reported teaching or feedback experiences, trainees perceived their main learning opportunities during outpatients or after‐hours placements. One participant reported that consultant attendance impeded teaching due to consultants having the role of ‘speaker’, minimising participation from others.

### 3.2 Supported Independence

A strong theme from trainees was the value of learning through active participation. This extended more broadly to outpatients and departmental presentations. Multiple trainees reported receiving more feedback when leading clinical encounters, although overall reports of feedback were low. The impact of using a bedside computer for electronic medical records was highlighted, noting limited learning opportunities and reduced active learning while being ward round scribe.

Independent learning was cited as another attractive feature, for example outpatient or evening roles where trainees worked autonomously with indirect supervision. These opportunities were infrequent during day shifts, with one trainee reporting their sole opportunity for independent patient assessment occurred while the fellow was on leave. While autonomy is important, one junior doctor described a period of excessive independence with no direct teaching and paucity of regular consultant ward rounds.

### 3.3 Clinical Learning Environment

The biggest barrier to ward round teaching for consultants was time. Interestingly, no junior trainees perceived time as a barrier to learning, instead offering methods to adapt to time limitations. Task management was a common theme from all participants, including feeling rushed, experiencing interruptions, high workload, and inconsistent after‐hours rounds.

Workplace culture was consistently raised by trainees, primarily around being included in the team and feeling safe to ask questions for learning. Usage of bedside computers on rounds also impacted trainees' ability to feel part of the team.

## 4 Discussion

Our study found increased consultant teaching and learner satisfaction after the introduction of the ward round education tool, despite poor perceived uptake of STIC by consultants. The magnitude of impact consultants have on workplace learning was emphasised by trainees and is consistent with previous studies [1, 13, 14]. Even consultants who self‐reported minimal behaviour change post‐STIC demonstrated increased engagement from trainee perspectives. Trainees reported increased learning value, indicating the positive influence of STIC or the implementation effort, even if not explicitly noted by consultants. Learner perception of the value of consultant‐led rounds was low at baseline; however, it substantially improved post‐STIC. Both the quantitative survey data and qualitative data indicate improvement, supporting the idea that the change was practically significant from the trainee perspective. This demonstrates how tools such as the STIC framework may alter vital consultant influence on ward‐round learning, even to a small degree, acknowledging that many contextual factors impact this experience. The difference between perceptions and the reality of the change may point to expectations of the degree of change anticipated when a new tool is introduced. This correlates with the initial STIC evaluation study, noting behaviour change takes time, with small incremental changes [9].

The role of learner‐centred teaching through active participation is reported in the literature [15], fitting with the social component of ward rounds [3]. This was highlighted in our study, with the highest learner satisfaction from self‐led rounds, and the common theme of desired autonomy. Communities of practice theory holds that supported participation in practice is central for students' learning [16]. The combination of supported independence via supervised clinical encounters, with targeted feedback, was reported by trainees as the preferred method of ward round learning. Consistent with improved consultant engagement post‐STIC, there were increased opportunities for trainees to lead despite increased consultant presence. This further indicates some impact of the tool and supports the benefit of active trainee participation. Supported independence in learner‐led encounters draws from the concept of engagement with new activities providing extension of the learner's knowledge via creation of new cognitive structures [17]. It is likely that individual learner factors also influenced learning. The overall low incidence of feedback reported in this study is consistent with other literature, with specific limitations being time, lack of supervised opportunities, and discomfort with feedback provision [2, 4]. Trainee suggestions for further improvement in ward round learning corresponded to techniques already incorporated in the STIC framework, including enhanced feedback delivery, suggesting a greater impact of the tool on learner satisfaction if implemented more robustly [9].

The clinical learning environment has been recognised as the place where theoretical components of the curriculum are integrated with the practical, and transformed into professional skills and attitudes through social, organisational and instructional support [16]. The differences in learner satisfaction between the two departmental teams at baseline and post‐implementation reflect culture, and the impact of affective and organisational environmental factors on learning [18]. Billett's work on workplace pedagogic practices expresses how a learner's workplace experience is a product of multiple historical–cultural and situational factors [19]. It was disappointing to find reflections from junior staff indicating fear of asking questions, lack of team inclusion and, consequently, concern regarding their perception by patients and families. Psychological safety is influenced by specific leadership behaviours, further substantiating the vital consultant role in education, and has been demonstrated to improve patient safety through trainee engagement and medical error reporting [20]. Supervisor approachability has been demonstrated to influence trainee decisions to seek clinical support and provision of quality patient care [21]. This study did not intend to specifically discuss nor address psychological safety, although principles of this are embedded in the tool. It is possible more change would eventuate through a multifaceted approach addressing both structure and culture. Yet, in practice, this was not possible and contextual readiness for different components of an intervention must be considered.

The implementation of change within healthcare organisations causes cultural shifts and can be complex and challenging [22]. Although learner suggestions for teaching improvement were already embedded in the STIC framework, there was difficulty altering consultant behaviour. True behaviour change requires enhanced implementation and persistent effort over a sustained period. Identification of personal values is useful to enable demonstration of shared values and an increased potential for engagement [23]. The evidence of baseline learner dissatisfaction in this study would likely provide additional consultant motivation in future implementation attempts.

Strengths of this study were its grounding in education theory, principally social constructivism, and prior research demonstrating the utility of the STIC tool in a separate environment [9]. The use of a mixed‐methods design increases the validity of this study and allows potential extrapolation of findings to alternative settings [24]. Limitations were the small cohort, single site and single junior doctor group. Furthermore, the dual role of the lead researcher as a consultant with hierarchical discrepancy may have limited anonymity and full disclosure. Reflexivity was considered, with confirmation bias a distinct risk, minimised by the use of a second researcher to review thematic analysis via an iterative process [25]. Although most departmental consultants participated, the opt‐in consent approach may have contributed to self‐selection bias. The restricted implementation period likely limited impact; however, the positive influence on learner satisfaction suggests the tool's potential impact with sustained implementation.

## 5 Conclusion

As ward rounds continue to be an indispensable part of clinical duties, ongoing strategies to improve teaching quality are required. The use of a ward round teaching tool has potential to improve learner satisfaction with teaching. This study highlights that in addition to a specific teaching focus, actions peripheral to teaching such as consultant engagement and creating a safe clinical learning environment are vital to improve learning.

## Ethics Statement

This study was approved by the Children's Health Queensland Human Research Ethics Committee (Reference: LNR/20/QCHQ/61830).

## Conflicts of Interest

The authors declare no conflicts of interest.

## Supporting information


Data S1.
