# Supplementary material for: The Use of a Ward Round Teaching Tool in a Paediatric Oncology Department
Source: J Paediatr Child Health. 2025 Jul 17;61(10):1582–8. doi: 10.1111/jpc.70155 (PMC12515269; doi:10.1111/jpc.70155)
Supplement: Supplementary file 1 — Data S1. [file JPC-61-1582-s001.docx]

### SUPPLEMENTAL FIGURE S1 Survey Questions for Junior Medical Participants

Survey questions for junior doctors assessing learner satisfaction with ward rounds across domains of the STIC tool, using a 5-point Likert scale and performed for 4 weeks pre- and 4 weeks post-implementation of the STIC tool.

Regarding TODAY’s ward round:

1. Who led the ward round?

| Consultant | Fellow | Registrar | Yourself |
| --- | --- | --- | --- |

1. I found it to be valuable for my learning

| 1 | 2 | 3 | 4 | 5 |
| --- | --- | --- | --- | --- |
| Not at all | A little | Neutral | Quite a lot | Very |

1. Was an agenda set?

| 1 | 2 | 3 | 4 | 5 |
| --- | --- | --- | --- | --- |
| Not at all | A little | Neutral | Quite clearly | Very clearly |

1. Were you given an opportunity to lead a clinical encounter?

| 1 | 2 | 3 | 4 | 5 |
| --- | --- | --- | --- | --- |
| Not at all | Rarely | Occasionally | A few times | Many times |

1. Were you provided with any feedback?

| 1 | 2 | 3 | 4 | 5 |
| --- | --- | --- | --- | --- |
| Not at all | Rarely | Occasionally | A few times | Many times |

1. Was the ward round closed, in terms of your understanding and learning?

| 1 | 2 | 3 | 4 | 5 |
| --- | --- | --- | --- | --- |
| Not at all | A little | Neutral | Quite clearly | Very clearly |

### SUPPLEMENTAL TABLE S1 Focus Group Questions

Key questions for junior and senior medical staff focus group and interviews.

| Question | Junior Doctors | Consultants |
| --- | --- | --- |
|  | Tell me your overall thoughts on teaching and learning in the unit? | Did you use the STIC framework and if so, how useful have you found it for ward round teaching? |
|  | How useful have you found the introduction of the STIC framework for ward round teaching? | Has any part of your ward round practice changed since the knowledge of the STIC framework, and please share an example? |
|  | Has the use of the STIC framework increased your learning on ward rounds, and please share an example? | Which part of the STIC framework did you find most useful? |
|  | Which part of the STIC framework did you find most useful? | Does using the STIC framework help you teach more effectively on ward rounds? |
|  | Has the use of the STIC framework increased the feedback you are provided with on ward rounds? | Has using the STIC framework increased the feedback you provide on ward rounds? |
|  | Is there any other way the STIC framework has impacted on your daily learning or job in general? | Has using the STIC framework taken up as much time as you expected? |
|  | What are the challenges to learning and what would help to improve teaching on rounds? | What are your thoughts about the barriers to teaching on ward rounds, and any suggestions for what would help improve this? |
